# Plant Growth-promoting Effects of Viable and Dead Spores of *Bacillus pumilus* TUAT1 on *Setaria viridis*

**DOI:** 10.1264/jsme2.ME21060

**Published:** 2022-01-27

**Authors:** Shin-ichiro Agake, Fernanda Plucani do Amaral, Tetsuya Yamada, Hitoshi Sekimoto, Gary Stacey, Tadashi Yokoyama, Naoko Ohkama-Ohtsu

**Affiliations:** 1 United Graduated School of Agriculture, Tokyo University of Agriculture and Technology, Saiwaicho 3–5–8, Fuchu-shi, Tokyo 183–8509, Japan; 2 Divisions of Plant Science and Technology and Biochemistry, University of Missouri, 1201 Rollins St., Columbia, MO 65201–4231, USA; 3 Joyn Bio, 37437 CA-16, Woodland, CA–95695; 4 Institute of Global Innovation Research, Tokyo University of Agriculture and Technology, Saiwaicho 3–5–8, Fuchu-shi, Tokyo 183–8509, Japan; 5 Institute of Agriculture, Tokyo University of Agriculture and Technology, Saiwaicho 3–5–8, Fuchu-shi, Tokyo 183–8509, Japan; 6 Faculty of Agriculture, Utsunomiya University, Minemachi 350, Utsunomiya-shi, Tochigi 321–8505, Japan; 7 Faculty of Food and Agricultural Sciences, Fukushima University, Kanayagawa 1, Fukushima-shi, Fukushima 960–1296, Japan

**Keywords:** *Bacillus pumilus*, PGPR, Spores, C4 model plant, *Setaria viridis*

## Abstract

Spores are a stress-resistant form of *Bacillus* spp., which include species that are plant growth-promoting rhizobacteria (PGPR). Previous studies showed that the inoculation of plants with vegetative cells or spores exerted different plant growth-promoting effects. To elucidate the spore-specific mechanism, we compared the effects of viable vegetative cells, autoclaved dead spores, and viable spores of *Bacillus pumilus* TUAT1 inoculated at 10^7^ CFU plant^–1^ on the growth of the C4 model plant, *Setaria viridis* A10.1. *B. pumilus* TUAT1 spores exerted stronger growth-promoting effects on *Setaria* than on control plants 14 days after the inoculation. Viable spores increased shoot weight, root weight, shoot length, root length, and nitrogen uptake efficiency 21 days after the inoculation. These increases involved primary and crown root formation. Additionally, autoclaved dead spores inoculated at 10^8^ or 10^9^ CFU plant^–1^ had a positive impact on crown root differentiation, which increased total lateral root length, resulting in a greater biomass and more efficient nitrogen uptake. The present results indicate that an inoculation with viable spores of *B. pumilus* TUAT1 is more effective at enhancing the growth of *Setaria* than that with vegetative cells. The plant response to dead spores suggests that the spore-specific plant growth-promoting mechanism is at least partly independent of symbiotic colonization.

Biofertilizers composed of plant growth-promoting rhizobacteria (PGPR) are an environmentally friendly strategy for enhancing plant growth while contributing to a reduction in the use of exhaustible chemical fertilizers, such as nitrogen, phosphorous, and potassium (Malusá *et al.*, 2012; Amundson *et al.*, 2015; Manning, 2015; Dilfuza and Adesemoye, 2016; Kumari *et al.*, 2019). The proposed mechanisms by which PGPR enhance plant growth include 1-aminocyclopropane-1-carboxylic acid deaminase production, phytohormone production, biological nitrogen fixation, antagonism against phytopathogens (*e.g.*, siderophores), the solubilization and mineralization of nutrients (P and K), enhanced resistance to abiotic stress, and the production of vitamin B (*e.g.*, biotin) (Glick *et al.*, 2007; Hayat *et al.*, 2010; Rajkumar *et al.*, 2010; Kudoyarova *et al.*, 2015; Pii *et al.*, 2015; Meena *et al.*, 2017; Yoshii *et al.*, 2019). We previously reported the development of the biofertilizer “Kikuichi” (Yume-bio; Asahi Agria) containing spore cells of *Bacillus pumilus* strain TUAT1, which enhanced rice growth and yield (Win *et al.*, 2018, 2019, 2020; Agake *et al.*, 2021). *B. pumilus* TUAT1 is a spore-forming, gram-positive, endophytic bacterium. The whole genome of this bacterium was sequenced by Okazaki *et al.* (2019), revealing the presence of genes for the production of indole-3-acetic acid (IAA), siderophores, and acetoin, which have been shown to promote plant growth and development. Ngo *et al.* (2019) demonstrated that an inoculation with *B. pumilus* TUAT1 spore cells promoted rice growth by increasing plant height, root weight, and shoot weight, whereas that with vegetative cells only altered plant height. Furthermore, an inoculation with the autoclaved, dead spore cells of *Bacillus* strains, such as *B. pumilus* TUAT1, JM52, MAFF118530, and MAFF301706, *B. altitudinis* JR4 and JR198, and *B. megaterium* MAFF301694 and MAFF520023 promoted rice growth even though their abilities to produce IAA, fix nitrogen, or solubilize phosphorous differed. Modifications to plant activities following an inoculation with autoclaved spores suggest the presence of substances common to spores that exert plant growth-promoting effects independent of the ability of bacteria to colonize plants (Habibi *et al.*, 2014; Seerat *et al.*, 2019).

*Setaria viridis* A10.1 is a C4 plant that responds to several PGPR (Pankievicz *et al.*, 2015; Faoro *et al.*, 2017; Alves *et al.*, 2019). The whole sequence of the reference genome of *S. viridis* was reported by Bennetzen *et al.* (2012) and a high quality assembly was recently released (Mamidi *et al.*, 2020). The findings of detailed investigations on C4 model plants may be translated to important crops, such as maize, sorghum, and sugarcane, which are being promoted as important feedstocks for bioenergy production (Roudier *et al.*, 2011; Koçar and Civaş, 2013).

Few studies have focused on differences in the plant growth-promoting effects of vegetative and spore cells (Posada *et al.*, 2016). Therefore, the present study investigated whether the spore-specific plant growth-promoting mechanism is effective on *Setaria* and examined the effectiveness of *B. pumilus* TUAT1 spores as a possible biofertilizer.

## Materials and Methods

### Plant materials

*S. viridis* A10.1 seeds were surface scarified using sulfuric acid with 95% (v/v) for 15‍ ‍min and then washed in current reverse osmosis (RO) water to break dormancy. Seeds were further treated with 1% (v/v) sodium hypochlorite with 0.1% (v/v) of Tween^®^ 20 for 3‍ ‍min followed by washing with sterilized RO water. Seeds were sown on agar plates containing Murashige and Skoog medium (1962) without any antibiotics and then incubated at 30°C for 1 day in the dark before being transferred to a growth chamber with photoperiod conditions of 16 h lights on 250‍ ‍μmol‍ ‍s^–1^‍ ‍m^–2^ at 28°C and 8 h lights off at 25°C for 4 days prior to treatment. Two germinated seedlings, which were not contaminated, were transplanted into 90.3‍ ‍cm^2^ of an autoclaved mixture of high fired calcined clay soil and vermiculite (3:1) (Pankievicz *et al.*, 2015). Transplanted seedlings were moved to an environmentally controlled room with photoperiod conditions of 16 h lights on 250‍ ‍μmol‍ ‍s^–1^‍ ‍m^–2^ and 8 h lights off at 28±2°C. Plants were irrigated with modified Hoagland’s solution containing 1‍ ‍mmol L^–1^ of KNO_3_ (Hoagland and Arnon, 1938) added to the bottoms of the pots once a week to 46% (v/v) of soil, and RO water was irrigated to maintain moisture on other days.

### Bacteria growth conditions

A wild-type strain and naturally acquired antibiotic-resistant strain (Rif^R^, Sm^R^) of *B. pumilus* TUAT1 reported in a previous study (Seerat *et al.*, 2019) were used as inoculants. The antibiotic-resistant strain was used to facilitate colony counting, while the wild-type strain was selected for the autoclaved dead spore inoculation test. Vegetative cells of *B. pumilus* TUAT1 were cultured in 300‍ ‍mL of trypticase soy (T-soy) broth in a 1-L Erlenmeyer flask at 180‍ ‍rpm at 30°C for 24 h. A high concentration of spore cells was obtained by culturing for 72 h in Difco sporulation medium (DSM) instead of T-soy broth (Nicholson and Setlow, 1990). Cells were collected by centrifugation and then washed with sterile water purified with a RO membrane several times and finally resuspended in sterile 0.85% (w/v) saline water. The high purities of vegetative cells and spores were confirmed by phase-contrast microscopic observations. The spore culture was incubated at 65°C for 1 h to kill vegetative cells and facilitate the transformation to spores. Dead spores were prepared by autoclaving at 121°C for 40‍ ‍min. We confirmed the mortality of all bacterial cells after autoclaving by an inoculation into T-soy broth and plating on T-soy and DSM agar plates. The colony-forming units (CFU) of inoculant cultures were confirmed by plating onto T-soy agar and optimal density (OD) 600.

### Inoculation of *S. viridis* with viable vegetative cells and viable spores

We initially tested inoculum forms of TUAT1, *i.e.*, viable vegetative cells, viable spores, and autoclaved spores at the same concentrations to elucidate the characteristics of each formulation. We used an antibiotic-resistant strain to quantify the degree of colonization of the roots. The concentration of the inoculant was adjusted to 10^7^ CFU mL^–1^ on the same day with the harvest of bacterial cells after confirming the correlation between CFU and OD_600_. Seedlings were transferred to soil and immediately inoculated with 1‍ ‍mL of each prepared inoculant, while control plants were treated with 0.85% (w/v) saline solution without bacteria. Seedlings were grown for 14 days in the environmentally controlled room. Ten healthy seedlings were randomly harvested to measure growth parameters, such as shoot length, shoot weight, and root weight. Each root was washed using tap water and mashed 9 times in sterile saline solution using a mortar and pestle, resulting in a 10-fold dilution, which was spread onto T-soy agar plates with 100‍ ‍mg L^–1^ of rifampicin and streptomycin (FUJIFILM Wako Pure Chemical) to count the total cell and spore cell numbers of *B. pumilus* TUAT1 (*n*=4 or 5, due to the lack of a sample). The number of spores was estimated by plate counting after heating the cell suspension at 65°C for 60‍ ‍min to kill vegetative cells. CFUs assessed after this heating step were then compared to the total number of viable CFUs counted before heating. The number of vegetative cells was calculated by subtracting the spore number from the total cell number (Spinosa *et al.*, 2000). The index of spores/vegetative cells was calculated using those numbers; a high number indicated that most of the colonizing bacteria formed spores.

### Inoculation of autoclaved dead spores onto S. viridis at 10^7^, 10^8^, and 10^9^ CFU plant^–1^

Inoculants in this experiment were prepared as follows: wild-type viable spores were suspended to a concentration of 10^9^ (ADS9) CFU mL^–1^ based on counting on T-soy agar. This suspension was autoclaved and diluted 10- and 100-fold to give final concentrations of 10^8^ (ADS8) and 10^7^ (ADS7), respectively. In the present study, the unit of dead spores is also described as CFU because inoculants were adjusted based on their colony-forming ability when they were viable spores before autoclaving. One milliliter of dead spore suspensions, viable spores at 10^7^ CFU mL^–1^, and saline solution as the control was used to inoculate seedlings as described above. Seedlings were cultivated under the same conditions as those described above, except for the cultivation period, namely, 21 days in the environmentally controlled room. After 21 days of cultivation, Soil Plant Analysis Development (SPAD) values were measured with a SPAD-502Plus instrument (Konica Minolta), which provides an *in situ* measurement of the leaf chlo­rophyll content (Süb *et al.*, 2015; Dunn *et al.*, 2018; Shibaeva *et al.*, 2020) using the second leaf from a randomly selected uppermost fully expanded leaf. Healthy plants were randomly harvested and the fresh weights of both the shoots and roots as well as shoot lengths were measured from a minimum of 20 healthy seedlings for each treatment. In addition, the randomized root samples of 10 seedlings were scanned using an Epson Perfection V700 Photo (Seiko Epson) and analyzed by WinRHIZO software ver. 2004 (Regent Instruments) to obtain information on the total root length, root area, root volume, primary and crown root lengths, the numbers of primary and crown roots, total lateral root (LR) length, and the number, density, and average length of lateral root. The roots of all 20 seedlings were dried at 80°C for 2 days, followed by dry weight measurements. Two dried shoot parts were combined as a single sample, and the nitrogen concentrations of 10 combined samples for each treatment were measured using the NC analyzer SUMIGRAPH NC TR-22 (Sumika Chemical Analysis Service).

### Statistical ana­lysis

Data collected for root and shoot weights, shoot length, phenotypic root parameters, and the nitrogen content of shoots were analyzed by Dunnett’s test and Tukey’s test with SPSS ver. 23. Significant differences in the level of bacterial colonization were calculated using the *t*-test.

## Results and Discussion

### Plant growth-promoting effects of viable spores

**Fig. 1.**
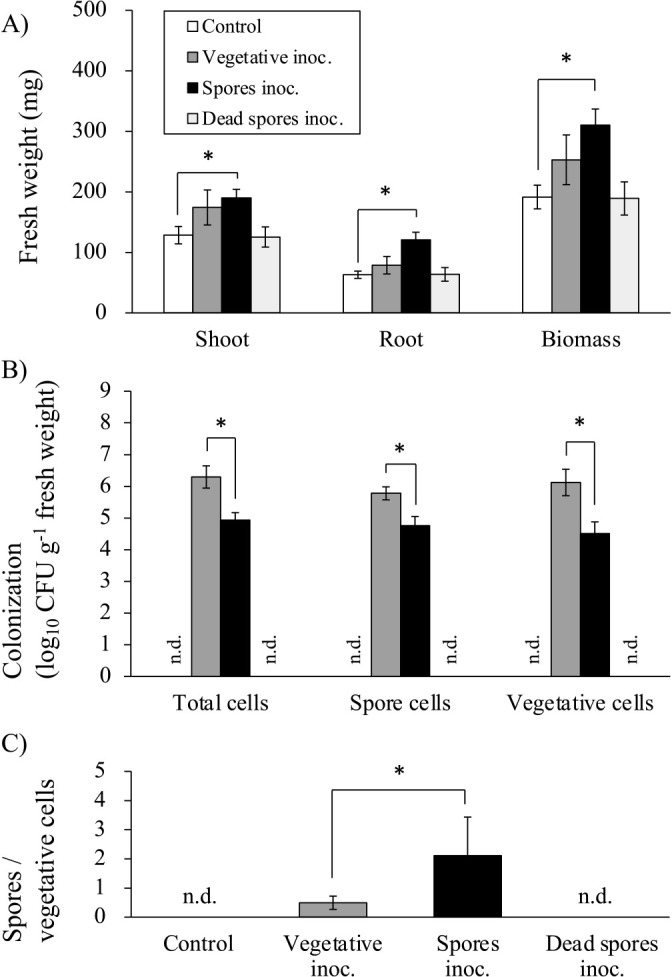
Growth parameters of seedlings 14 days after inoculation and information on colonized TUAT1. A) The graph shows the means of shoot, root, and biomass fresh weights following an inoculation with 0.85% (w/v) of saline solution (control) and 10^7^ CFU plant^–1^ of viable vegetative cells (dark gray bars), viable spores (black bars), and autoclaved, dead spores (light gray bars). (*n*=10). B) The number of colonized total cells, spores, and vegetative cells on the roots are shown (*n*=4 or 5), and C) the ratio of colonized spores to vegetative cells is shown (*n*=4 or 5). Error bars indicate standard errors. Asterisks indicate significant differences (*P*<0.05) by Dunnett’s test for fresh weights (a), and the *t*-test between treatments (b and c). inoc., inoculation. n.d., not detected.

The inoculation with 10^7^ CFU plant^–1^ viable spores of *B. pumilus* resulted in significantly higher shoot, root, and total fresh weights in *S. viridis* plants than in control plants after 14 days (Fig. 1A). The growth ratios of shoots inoculated with viable vegetative cells, spores, and dead spores relative to the control were 1.36, 1.48, and 0.98, respectively. Shoots inoculated with viable spores showed a significant difference from the control in Dunnett’s test, but not in Tukey’s test. The growth ratios of roots inoculated with vegetative cells, spores, and dead spores relative to the control were 1.25, 1.91, and 1.01, respectively, while those of the biomass were 1.32, 1.62, and 0.99, respectively. As shown in Fig. 1B, the numbers of total cells, spores, and vegetative cells colonizing plant roots were higher with the vegetative cell inoculant (dark gray bars) than with the viable spores inoculant (black bars), irrespective of the plant growth response. These results are consistent with previous findings showing that the bacterial colonization of roots did not correlate with the degree of plant growth promotion (Alves *et al.*, 2019). Regardless of the inoculum forms, vegetative cells and spores were detected on roots. A higher quantity of spores (5.98×10^5^ CFU g^–1^) was obtained in roots with the vegetative cell inoculation than with the viable spores inoculation. The transformation between spores and vegetative cells in the root or rhizosphere was demonstrated by the presence of vegetative cells and spores in the spore- and vegetative cell-inoculated roots, respectively. To obtain a more detailed understanding of the primary forms in inoculated roots, the index of colonizing spores/vegetative cells was assessed (Fig. 1C). The index for the vegetative inoculation 14 days after transplanting was 0.49, while that for the viable spores inoculation was 2.11, indicating that the abundance of colonizing spores was higher in viable spores-inoculated roots. This is consistent with the findings by Seerat *et al.* (2019). i.e., *B. pumilus* TUAT1, colonizing the rhizoplane and endosphere of rice cultivar “Hitomebore” inoculated with its viable spores, was mostly spores in morphology upon root growth promotion. Therefore, the spore-specific plant growth-promoting effects of TUAT1 may be dependent on a high ratio of spores to vegetative cells, but not on the total number of bacteria colonizing the root. In contrast, Posada *et al.* (2016) reported that in comparisons with a spore inoculant of *B. subtilis* strain EA-CB0575 at 1×10^7^ CFU mL^–1^, an inoculation with 1×10^7^ CFU mL^–1^ of its vegetative cells significantly increased the shoot length and dry biomass of banana plants; however, both inoculants significantly increased plant growth over that of uninoculated controls.

### Autoclaved dead spores promote S. viridis plant growth

**Fig. 2.**
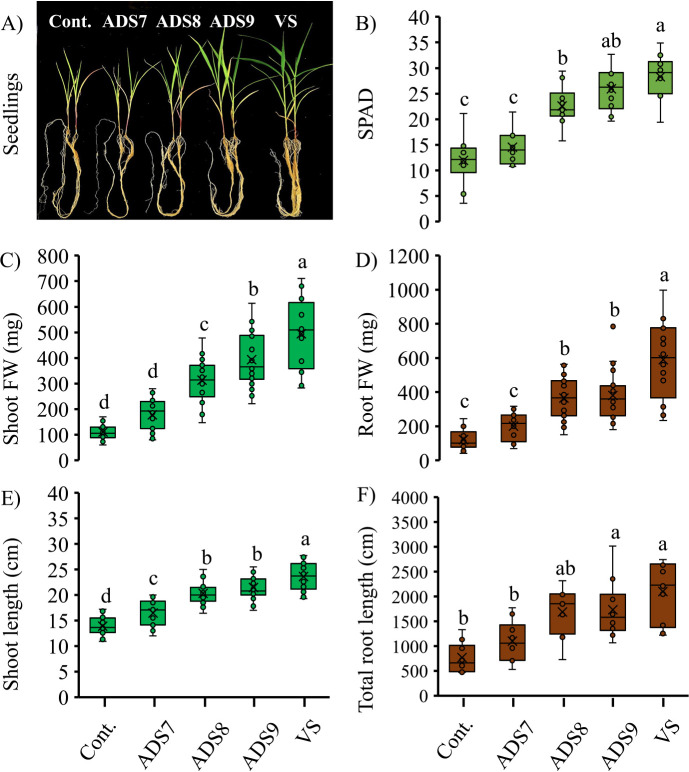
Growth of *Setaria viridis* A10.1. 21 days after transplanting. A) The image shows the morphologies of seedlings inoculated by saline solution alone as the control (Cont.), 10^7^ CFU plant^–1^ of autoclaved dead spores (ADS7), 10^8^ CFU plant^–1^ of autoclaved dead spores (ADS8), 10^9^ CFU plant^–1^ of autoclaved dead spores (ADS9), and 10^7^ CFU plant^–1^ of viable spores (VS) (*n*=20). Each box plot shows the measured parameters as B) SPAD values (*n*=10), C) shoot fresh weight (*n*=20), D) root fresh weight (*n*=20), E) shoot length (*n*=20), and F) total root length (*n*=10). Letters indicate a significant different calculated by Tukey’s test and the Tukey-Kramer method.

To verify the cell concentration-dependent effects of autoclaved spores on the growth of *Setaria*, the concentrations of inoculants were increased from 10^7^ CFU plant^–1^ (ADS7) to 10^8^ (ADS8) and 10^9^ (ADS9). Strong plant growth-promoting effects by the ADS9 and viable spores (VS) inoculants were observed after 21 days (Fig. 2A). The non-inoculated control and ADS7-inoculated seedlings showed nutrient deficiency symptoms that were not visible in plants after the ADS8 or ADS9 inoculation. The SPAD values of each treatment were measured to monitor the chlorophyll content of the shoot and the results obtained are shown in Fig. 2B. Seedlings inoculated with VS registered the highest chlorophyll content in all treatments; a 137% increase from control plants, although this value was not significantly different from plants inoculated with ADS9. Seedlings inoculated with ADS8 or ADS9 also showed higher SPAD values than control or ADS7-inoculated plants, with increases of 89 and 117%, respectively, from control plants. The VS inoculation led to a 344% increase in shoot fresh weight and 387% increase in root fresh weight over the control at 21 days (Fig. 2C and D). The ADS8 and ADS9 inoculations increased shoot and root fresh weights, with the former showing increases of 181% in shoots and 201% in roots and the latter showing increases of 253 and 214%, respectively. The shoot fresh weights of plants inoculated with ADS9 were significantly higher than those inoculated with ADS8, while no significant differences were observed in root fresh weights. Shoot and root fresh weights were both significantly lower after the inoculation with ADS9 than with VS. ADS7 did not significantly increase shoot or root fresh weights, which was similar to the effects of the autoclaved spores of the antibiotic-resistant strain 14 days after the inoculation (Fig. 1A). However, the inoculation with ADS7 resulted in a significantly higher (19%) plant shoot length than that of the control 21 days after transplanting (Fig. 2E). Similar effects on shoot length were noted using the ADS8, ADS9, and VS inoculations, with increases of 47, 55, and 70%, respectively, from that of the control. In addition, the ADS9 and VS inoculations resulted in a significantly higher total root length (primary root, crown roots, and lateral root) than those in the control and plants inoculated with ADS7 (Fig. 2F). The growth-promoting effects of dead spores on shoot weight were dependent on concentration; however, this did not appear to be the case for root fresh weight. The present results indicated that the inoculant concentration needed to increase root fresh weight was 10^8^ CFU plant^–1^ for dead spores. The inoculation of seedlings with 10^7^ CFU plant^–1^ of dead spores significantly increased shoot length, but not SPAD values, shoot and root weights, or total root length. In the literature, it is common to assume that dead bacterial cells have no plant growth-promoting effects and, thus, dead cell preparations were used as negative controls in some studies (Kuan *et al.*, 2016). The present results suggest that this assumption may not be valid under all conditions and, at least for *Setaria*, clear plant growth-promoting effects were detected following an inoculation with dead spore suspensions.

**Fig. 3.**
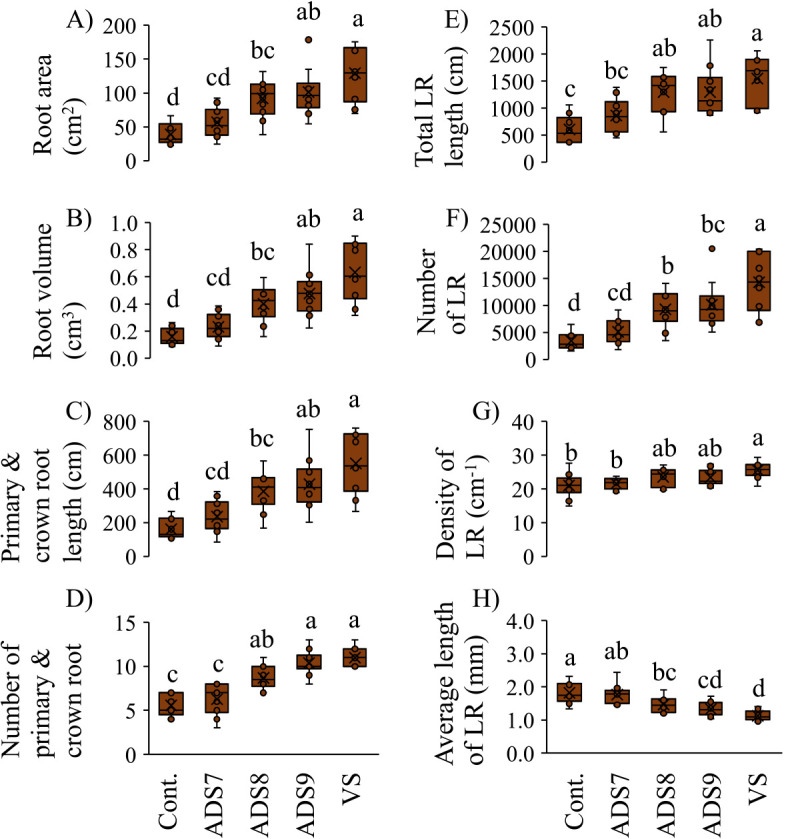
Morphological ana­lysis of roots 21 days after transplanting. Box plots show the parameters of each root analyzed by a root scanner: A) total surface area, B) total volume, C) total length of the primary and crown roots, D) number of primary and crown roots, E) total length of the lateral roots (LR), F) total number of LR, G) density of LR, and H) average length of LR. Seedlings were inoculated with saline solution alone as the control (Cont.), 10^7^ CFU plant^–1^ of autoclaved dead spores (ADS7), 10^8^ CFU plant^–1^ of autoclaved dead spores (ADS8), 10^9^ CFU plant^–1^ of autoclaved dead spores (ADS9), and 10^7^ CFU plant^–1^ of viable spores (VS). Letters indicate a significant difference calculated by Tukey’s test and the Tukey-Kramer method (*n*=10).

The results of the morphological ana­lysis of roots are shown in Fig. 3A, B, C, D, E, F, G, and H. Among all the treatments tested, the total root surface area and volume showed the greatest increases in plants inoculated with VS (Fig. 3A and B). These parameters did not significantly differ between plants inoculated with ADS9 and VS. Primary roots representative of each treatment were analyzed along with crown roots because their root diameters were similar (Fig. 3C and D). The total length of the primary root and crown roots was significantly shorter in plants inoculated with ADS8 than in those inoculated with VS, and was not longer than that in plants inoculated with ADS7. No significant differences were observed in the total number of primary root and crown roots between ADS8- and VS-treated plants; however, it was significantly higher than in plants inoculated with ADS7. These results suggest that autoclaved dead spores enhanced the differentiation of crown roots instead of elongating the primary and crown roots. No significant differences were noted in the total lateral root length between plants receiving different concentrations of autoclaved spores (Fig. 3E). The inoculation with VS markedly increased the total number of lateral root, whereas that with autoclaved killed spores, regardless of the concentration, exerted weaker effects than the VS inoculant (Fig. 3F). The densities of lateral root between different autoclaved spore concentrations were not significantly different (Fig. 3G), which was similar to the results obtained for total lateral root length. The VS inoculation reduced average lateral root length, and this decrease was also observed with the ADS8 and ADS9 inoculants (Fig. 3H). Ngo *et al.* (2019) reported that the inoculation of rice with viable spores increased the number and density of lateral root, while that with viable vegetative cells did not. Furthermore, the inoculation with viable spores suppressed average lateral root length, whereas that with vegetative cells increased mean lateral root length. These findings are consistent with the present results showing that the inoculation of *Setaria* with VS increased the number and density of lateral root (Fig. 3F‍ ‍and G) and reduced their average‍ ‍length (Fig. 3H). However, the inoculation with autoclaved dead spores appeared to exert stronger effects on the total length and number of lateral root with no observable effect on their‍ ‍density or average length enhancement (Fig. 3E, F, G, and H). Collectively, these results suggest that the autoclaved spore inoculant mainly affected the differentiation of crown roots, resulting in increases of lateral roots length, while viable spores appeared to alter the differentiation of lateral root (*i.e.*, density).

**Fig. 4.**
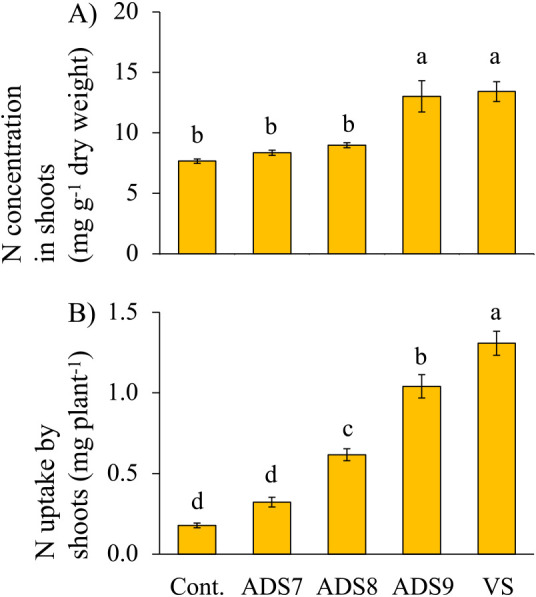
Graphs of nitrogen concentrations and uptakes. The graphs show A) nitrogen concentrations in shoots and B) nitrogen uptake by shoots inoculated with saline solution alone as the control (Cont.), 10^7^ CFU plant^–1^ of autoclaved dead spores (ADS7), 10^8^ CFU plant^–1^ of autoclaved dead spores (ADS8), 10^9^ CFU plant^–1^ of autoclaved dead spores (ADS9), and 10^7^ CFU plant^–1^ of viable spores (VS). Letters indicate a significant difference calculated by Tukey’s test and the Tukey-Kramer method (*n*=10). Error bars indicate standard errors. conc., concentration.

In addition to our plant growth measurements, we assessed nitrogen (N) content in and N uptake by shoots for all inoculant treatments, as shown in Fig. 4A and B, respectively. The mean N contents of plants inoculated with VS and ADS9 were 13.4 and 13.0‍ ‍mg g^–1^, respectively, and these values were significantly higher than those in uninoculated control plants or plants inoculated with ADS7 and ADS8. N uptake by shoots inoculated with VS was 1.3‍ ‍mg N plant^–1^, which was approximately six-fold higher than that in control plants and the strongest response to any of the treatments (Fig. 4B). N uptake by ADS9-inoculated plants was 1.0‍ ‍mg N plant^–1^, which was significantly less than that by plants inoculated with VS. The ADS8 inoculation increased N uptake over that by control plants, whereas no effect was observed with the ADS7 inoculant. *B. pumilus* TUAT1 lacks nitrogen fixation genes (Okazaki *et al.*, 2019), and Win *et al.* (2018) previously suggested that the increase observed in N uptake by this strain on rice was due to other mechanisms. Therefore, increased SPAD values and nitrogen concentrations in *Setaria* plants inoculated with VS may be due to the promotion of root growth. The inoculation with autoclaved dead spores also increased the plant N content, which may also be attributed, at least in part, to its effects on root parameters.

Ngo *et al.* (2019) suggested that an inoculation with *B. pumilus* TUAT1 spores promoted crown root formation and elongation by enhancing the production of auxin (Ngo *et al.*, 2019). The elongation of crown roots and enhanced crown root differentiation were the most strongly affected by the VS inoculation (Fig. 3C). However, the inoculation with autoclaved killed spores also increased the crown root number and length even though bacterial metabolism was clearly not occurring. One possibility is that metabolites leak from spores during autoclaving and have the ability to impact plant growth. However, we were unable to confirm whether these compounds were minerals, amino acids, sugars, or oligomers from the spore and/or insoluble compounds, such as the spore membrane and coat residue (Hitchener and Egan, 1977; Tatsuguchi *et al.*, 1983; Tsuchido *et al.*, 1985; Tsuchido and Takano, 1988; Woo *et al.*, 2000; Russell, 2003; Li *et al.*, 2018). Candidate compounds with low molecular weights may include IAA, acetoin, and siderophores, the biosynthetic genes for which are encoded within the published genome of *B. pumilus* TUAT1 (Okazaki *et al.*, 2019). IAA is an important plant growth regulator and its effects are dependent on its concentration (Bonner and Koepfli, 1939; Ryu *et al.*, 2003; Gago *et al.*, 2009). IAA is light sensitive and poorly soluble in water (1.5‍ ‍mg mL^–1^ at 20°C; Shiu *et al.*, 1990). However, previous studies reported that the optimal concentration of dosed IAA to promote maize growth was 10^–5^ mol L^–1^ and demonstrated that it was heat-stable to autoclaving in neutral solution (Yamakawa *et al.*, 1979; Polak *et al.*, 2011). Acetoin is a soluble and volatile compound that is indispensable for the metabolism of the sporulation-related response regulator Spo0A. *Bacillus cereus* has been shown to utilize acetoin for the formation of poly-3-hydroxybutyrate (PHB), which is also a candidate as a higher molecular weight substance for spore-specific plant growth promotion, as described below (Kominek and Halvorson, 1965; Quisel *et al.*, 2001). Siderophores are soluble and not volatile and, thus, appear to be tolerant to autoclaving. *Bacillus* spp. produce catecholate siderophores (*e.g.*, bacillibactin and petrobactin) to enhance ferric iron uptake, and the biosynthesis and uptake genes for these siderophores were commonly found in several *Bacillus* spp. (*e.g.*, *B. anthracis*, *B. thuringiensis*, and *B. cereus*) (Dertz *et al.*, 2006; Hotta *et al.*, 2010). In addition, the sporulation of *Bacillus subtilis* was promoted by external bacillibactin (Grandchamp *et al.*, 2017). They also play an important role in plant colonization by bacteria (Khan *et al.*, 2017; Yi *et al.*, 2018). Possible candidate compounds with a higher molecular weight include PHB, which is insoluble in water, and peptidoglycan (PGN). PHB was identified as an important substance for the growth promotion of *S. viridis* by *Herbaspirillum seropedicae*, which is a gram-negative endophyte and nitrogen-fixing bacterium. Mutants of *H. seropedicae* with dysfunctional PHB production were not defective for *Setaria* root colonization; however, depending on the mutant analyzed, they affected the promotion of plant growth (Alves *et al.*, 2019). PHB of *B. thuringiensis* has been shown to accumulate during vegetative growth and is then catabolized before sporulation (Wang *et al.*, 2016). Therefore, we assume that PHB was not related to the autoclaved dead spore inoculation, but played a role in the viable bacterial or gram-negative bacterial inoculation and promotion of plant growth. On the other hand, PGN is a primary component of the bacterial cell wall and has been shown to elicit plant pathogen defense responses (Willmann *et al.*, 2011; Liu *et al.*, 2012, 2014). The structure of PGN in autoclaved spores may differ (*e.g.*, bigger or denatured) from those digested by lysozyme, and compositional differences depending on the bacterial species have been reported. Furthermore, the structure of PGN differs between spore and vegetative cells. Germ cell wall PGN, which is the initial wall of the outgrowing spore, is chemically different than thicker outer cortex layer PGN, which is decomposed during germination (Popham and Bernhards, 2015). Therefore, PGN may be a spore-specific compound.

The present results demonstrated that the inoculation of the C4 model plant, *S. viridis* A10.1 with *B. pumilus* TUAT1 spores significantly promoted plant growth. Plant growth was also promoted by the inoculation with autoclaved dead spores at concentrations of 10^8^ and 10^9^ CFU plant^–1^. The autoclaved spore inoculation promoted crown root formation, resulting in increases in total lateral root length, which may have enhanced nitrogen uptake. Since killed spores promoted plant growth, bacterial symbiotic colonization was not necessary for the spore-specific mechanism. We hypothesize that unknown plant growth-promoting substances leak from autoclaved spores and may be responsible for the effects observed in the present study.

## Citation

Agake, S., Plucani do Amaral, F., Yamada, T., Sekimoto, H., Stacey, G., Yokoyama, T., and Ohkama-Ohtsu, N. (2022) Plant Growth-promoting Effects of Viable and Dead Spores of *Bacillus pumilus* TUAT1 on *Setaria viridis*. *Microbes Environ ***37**: ME21060.

https://doi.org/10.1264/jsme2.ME21060
